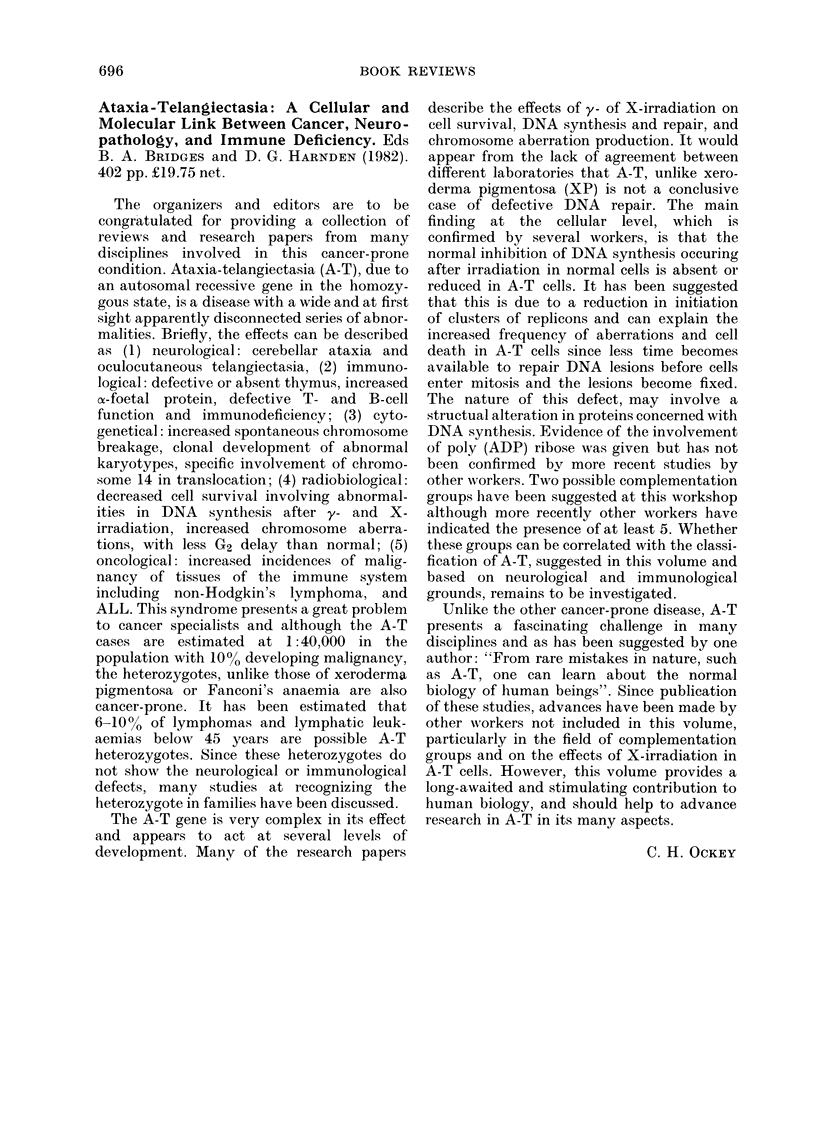# Ataxia-Telangiectasia: A Cellular and Molecular Link Between Cancer, Neuropathology, and Immune Deficiency

**Published:** 1982-10

**Authors:** C. H. Ockey


**Ataxia-Telangiectasia: A Cellular and Molecular Link Between Cancer, Neuropathology, and Immune Deficiency.** Eds B. A. Bridges and D. G. Harnden (1982). 402 pp. £19.75 net.

The organizers and editors are to be congratulated for providing a collection of reviews and research papers from many disciplines involved in this cancer-prone condition. Ataxia-telangiectasia (A-T), due to an autosomal recessive gene in the homozygous state, is a disease with a wide and at first sight apparently disconnected series of abnormalities. Briefly, the effects can be described as (1) neurological: cerebellar ataxia and oculocutaneous telangiectasia, (2) immunological: defective or absent thymus, increased $\alpha$-foetal protein, defective T- and B-cell function and immunodeficiency; (3) cytogenetical: increased spontaneous chromosome breakage, clonal development of abnormal karyotypes, specific involvement of chromosome 14 in translocation; (4) radiobiological: decreased cell survival involving abnormalities in DNA synthesis after $\gamma$- and X-irradiation, increased chromosome aberrations, with less $G_2$ delay than normal; (5) oncological: increased incidences of malignancy of tissues of the immune system including non-Hodgkin's lymphoma, and ALL. This syndrome presents a great problem to cancer specialists and although the A-T cases are estimated at 1:40,000 in the population with 10% developing malignancy, the heterozygotes, unlike those of xeroderma pigmentosa or Fanconi's anaemia are also cancer-prone. It has been estimated that 6–10% of lymphomas and lymphatic leukaemias below 45 years are possible A-T heterozygotes. Since these heterozygotes do not show the neurological or immunological defects, many studies at recognizing the heterozygote in families have been discussed.

The A-T gene is very complex in its effect and appears to act at several levels of development. Many of the research papers describe the effects of $\gamma$- of X-irradiation on cell survival, DNA synthesis and repair, and chromosome aberration production. It would appear from the lack of agreement between different laboratories that A-T, unlike xeroderma pigmentosa (XP) is not a conclusive case of defective DNA repair. The main finding at the cellular level, which is confirmed by several workers, is that the normal inhibition of DNA synthesis occuring after irradiation in normal cells is absent or reduced in A-T cells. It has been suggested that this is due to a reduction in initiation of clusters of replicons and can explain the increased frequency of aberrations and cell death in A-T cells since less time becomes available to repair DNA lesions before cells enter mitosis and the lesions become fixed. The nature of this defect, may involve a structual alteration in proteins concerned with DNA synthesis. Evidence of the involvement of poly (ADP) ribose was given but has not been confirmed by more recent studies by other workers. Two possible complementation groups have been suggested at this workshop although more recently other workers have indicated the presence of at least 5. Whether these groups can be correlated with the classification of A-T, suggested in this volume and based on neurological and immunological grounds, remains to be investigated.

Unlike the other cancer-prone disease, A-T presents a fascinating challenge in many disciplines and as has been suggested by one author: "From rare mistakes in nature, such as A-T, one can learn about the normal biology of human beings". Since publication of these studies, advances have been made by other workers not included in this volume, particularly in the field of complementation groups and on the effects of X-irradiation in A-T cells. However, this volume provides a long-awaited and stimulating contribution to human biology, and should help to advance research in A-T in its many aspects.

C. H. Ockey